# Moral Distress and Intention to Leave Current Position Among Registered Dietitians: A Cross‐Sectional Study

**DOI:** 10.1111/jhn.70368

**Published:** 2026-10-25

**Authors:** Susanna Pohjola, Åsa Grauman, Anna T. Höglund, Elin Lövestam

**Affiliations:** ^1^ Department of Public Health and Caring Sciences Uppsala University Uppsala Sweden; ^2^ Centre for Research Ethics & Bioethics Uppsala University Uppsala Sweden; ^3^ Department of Food Studies, Nutrition and Dietetics Uppsala University Uppsala Sweden

**Keywords:** cross‐sectional study, healthcare, moral distress, registered dietitian, turnover intention

## Abstract

**Background:**

Moral distress has been widely studied across healthcare professions, but registered dietitians [RDs] remain underrepresented in this research. The aims of this study were to describe moral distress among RDs, explore differences across healthcare contexts and examine the association between moral distress and current intention to leave a position.

**Methods:**

A cross‐sectional study was conducted using a web‐based survey among RDs working in primary and specialist care in Sweden. Moral distress was measured using the Measure of Moral Distress for Healthcare Professionals (MMD‐HP; total score range 0–432), including four dimensions: system level, patient level, team/staff‐level and team/patient‐level moral distress. Descriptive and regression analyses were employed to examine moral distress and its association with intention to leave the current position. The study was approved by the Swedish Ethical Review Authority (reference number 2023‐03151‐01 and amendment 2026‐00124‐02).

**Results:**

A total of 211 RDs were included. Participants reported a median total moral distress score of 71 (44–113). System‐level moral distress showed the highest standardised scores among the four dimensions of the MMD‐HP. No significant differences in total moral distress were observed between primary and specialist care. Higher levels of moral distress were associated with an increased likelihood of considering leaving the current position (OR = 1.016, 95% CI 1.008–1.023, *p* < 0.001).

**Conclusion:**

The findings contribute to the limited literature on moral distress among RDs and suggest that moral distress may be relevant not only as an ethical issue but also in relation to turnover intention within dietetic practice.

## Practitioner Points

- Higher levels of moral distress among RDs were associated with an increased likelihood of considering leaving the current position.
- Among the four MMD‐HP dimensions, system‐level moral distress showed the highest standardised scores, followed by team/patient‐, patient‐ and team/staff‐related moral distress.
- These findings highlight moral distress as a relevant issue for dietetic practice and support further research to identify strategies to address moral distress in clinical care.

## 1 Introduction

Moral distress refers to the distress that may arise when healthcare professionals are constrained from acting in accordance with what they believe to be ethically appropriate. Research has linked moral distress to a range of negative outcomes, including intentions to leave a position. Despite working in healthcare settings characterised by ethical challenges, resource constraints and complex treatment decisions, registered dietitians (RDs) remain underrepresented in this field of research. To our knowledge, no previous study has quantitatively examined moral distress and its association with intention to leave among RDs.

The dietetic profession is a relatively young healthcare profession with a broad and multifaceted role, where ethical frameworks are still evolving [1]. In 2024, there were 1427 RDs working in healthcare in Sweden [2]. Registered dietitians therefore represent a relatively small professional group within healthcare. In studies including multiple professions (e.g., [3]), RDs are rarely reported as a separate professional group, making it difficult to determine to what extent their perspectives are represented in the literature on moral distress.

In both primary and specialist care, nutritional assessment and treatment recommendations involve recurring ethical considerations influenced by the patient's life situation, team priorities and organisational conditions. In specialist care, ethical issues often involve decisions about enteral and parenteral nutrition, life‐sustaining treatment and nutritional support in palliative care [4, 5, 6, 7, 8, 9]. Research in primary care is more limited but shows that professionals deal with ethical considerations related to prioritisation, uncertainty in decision‐making, time pressure and taking patients' values and life situations into account [10, 11, 12, 13, 14]. Previous qualitative studies among dietitians have also highlighted challenges related to continuity of nutrition care, communication across services, referral pathways and access to community dietetic services [15]. Such organisational and interprofessional challenges may represent important sources of moral distress in dietetic practice.

Moral distress has been studied across several healthcare professions and is commonly defined as the stress that arises when healthcare professionals are repeatedly constrained from acting in accordance with their values. This may lead to feelings of not being able to provide adequate care, frustration and powerlessness [16, 17, 18, 19, 20, 21, 22]. The concept originates from nursing research and has predominantly been studied among nurses in hospital settings [20, 21].

More recent reviews show that moral distress occurs across healthcare contexts, with recurring themes including poor ethical climate, limited collaboration, lack of support and communication‐related challenges. Moral distress has also been linked to burnout, reduced wellbeing, lower job satisfaction and intentions to leave a position [3, 23, 24].

Against this background, the present study aims to describe moral distress among RDs in Sweden, explore differences across healthcare contexts and examine its association with current intention to leave a position. An exploratory psychometric analysis was conducted due to the limited use of the MMD‐HP among RDs.

### 1.1 Research Questions

- What are the levels and patterns of moral distress among RDs, in terms of total levels, subscales and individual items?
- Do levels of moral distress differ across healthcare contexts?
- Is there an association between moral distress and current intention to leave a position, after adjustment for healthcare context, professional experience and education level?

## 2 Materials and Methods

### 2.1 Study Design

This cross‐sectional study was conducted using a web‐based survey among RDs working in primary and specialist care in Sweden. The study was carried out within the Swedish healthcare context, where care is predominantly publicly funded but delivered by both public and private providers. Primary care was defined as healthcare services providing open care without restriction regarding diseases, age groups or patient categories [25], whereas specialist care was defined as healthcare services requiring more specialised interventions than can be provided in primary care [26].

The study is reported in accordance with the STROBE guidelines for cross‐sectional studies [27]. The completed checklist is available in Table S1.

### 2.2 Participants, Recruitment and Data Collection

The target population consisted of RDs engaged in clinical patient care in Sweden. The study was exploratory and based on a convenience sample. No sample size calculation was performed; recruitment aimed to reach as many eligible RDs as possible during the data collection period. Participants were initially recruited via the newsletter of the Swedish Association of Registered Dietitians (DRF). As RDs working in primary care were underrepresented, recruitment was expanded to include additional DRF‑related channels, including social media groups, as well as the authors' professional networks.

Inclusion criteria were employment as an RD, clinical patient care as the primary occupational role and employment in primary or specialist care. These criteria were verified through survey logic, and participants who did not meet them were excluded from further participation.

For analyses of moral distress, participants who discontinued the survey before providing sufficient data to calculate at least one MMD‐HP subscale score were excluded. For psychometric analyses, all participants who met the inclusion criteria were included regardless of the extent of completed MMD‐HP data.

Study data were collected and managed using REDCap electronic data capture tools hosted at Uppsala University [28, 29]. Data collection took place between March and May 2026 over a total of 10 weeks.

### 2.3 Measures

In this study, moral distress was measured using the Measure of Moral Distress for Healthcare Professionals (MMD‑HP). The instrument was developed to capture situations and circumstances associated with moral distress at patient, team and system levels [30]. It has been used in several clinical contexts and has been translated, culturally adapted and validated in multiple countries, including Sweden [31, 32, 33, 34, 35, 36, 37]. The MMD‑HP consists of 27 items describing situations in healthcare that may give rise to moral distress. Each item is rated for frequency and intensity on separate 0–4 scales, and item scores are calculated as the product of frequency and intensity (range 0–16). Total moral distress is calculated as the sum of all item scores (range 0–432). Subscale scores were calculated according to the established four‐factor structure of the instrument: system level (8 items; 0–128), patient level (6 items; 0–96), team/staff level (7 items; 0–112) and team/patient level (6 items; 0–96). To facilitate comparisons across subscales containing different numbers of items, standardised subscale scores were calculated by dividing each subscale score by the number of items in the respective subscale, resulting in scores ranging from 0 to 16.

Three cognitive interviews were conducted using a think‐aloud approach with RDs to assess the relevance and comprehensibility of the survey [38, 39]. The survey was subsequently pilot tested with regard to instructions, structure and functionality. Minor adjustments were made to instructions and wording, but no changes were made to the MMD‐HP items.

In addition to the MMD‑HP items, the survey included two supplementary questions concerning participants' experiences of leaving or considering leaving a position due to moral distress and their current intention to leave. Similar questions have been employed in previous MMD‐HP studies to examine the association between moral distress and intention to leave a position [40, 41, 42]. In the present study, the variable reflecting current intention to leave was included in the statistical analyses, while the variable capturing prior experiences was used for descriptive purposes.

Participants could also describe up to three additional situations perceived as giving rise to moral distress and rate their frequency and intensity (see Table S2).

Sociodemographic and professional variables were collected, including age, sex, education level, years of professional experience, healthcare context and patient groups encountered in clinical practice.

### 2.4 Data Analysis

The distribution of moral distress (total score and subscales) was assessed using histograms, boxplots and the Shapiro–Wilk test. As the scores were not normally distributed, non‐parametric tests were used for group comparisons. Descriptive statistics were presented as medians and interquartile ranges for continuous variables and as frequencies and percentages for categorical variables.

Analytical samples were defined based on available MMD‑HP data. Calculation of the total score required complete data for all 27 items, whereas subscale scores required complete data within each respective subscale. Participants with partial data could therefore be included in some analyses but not others, resulting in varying analytical sample sizes.

Missing data were handled using listwise deletion for total and subscale scores. No imputation was performed, as the MMD‑HP is based on a product score (frequency × intensity) and includes heterogeneous items, which limits the feasibility of reliable imputation. For psychometric analyses, all available item‐level data were employed.

### 2.5 What Are the Levels and Patterns of Moral Distress Among RDs, in Terms of Total Levels, Subscales and Individual Items?

Moral distress was summarised using descriptive statistics for total scores, subscale scores and individual items, presented as medians and interquartile ranges. Subscales were used descriptively to identify areas contributing most to moral distress and were ranked based on standardised subscale scores, while items were ranked based on item scores.

### 2.6 Do Levels of Moral Distress Differ Across Healthcare Contexts?

Differences in total and subscale scores between RDs in primary and specialist care were analysed using Mann–Whitney U tests.

### 2.7 Is There an Association Between Total Moral Distress and Current Intention to Leave a Position, After Adjustment for Healthcare Context, Professional Experience and Education Level?

The association between total moral distress and current intention to leave a position was analysed using binary logistic regression, with intention to leave the current position (yes/no) as the outcome. Healthcare context, years of professional experience and education level were included as potential confounders because they may be related to both moral distress and intention to leave a position. Model fit, discrimination and linearity in the logit were assessed as part of the regression diagnostics.

### 2.8 Free‐Text Responses

Free‐text responses were summarised descriptively to illustrate and contextualise the quantitative findings (see Table S2).

### 2.9 Ethical Considerations

The study was approved by the Swedish Ethical Review Authority (reference number 2023‐03151‐01 and amendment 2026‐00124‐02). Participation was voluntary, and informed consent was obtained through completion of the survey. Data were handled confidentially and analysed at the group level.

## 3 Results

A total of 211 RDs were included in the study. A response rate could not be calculated, as the total number of potential participants reached through recruitment is unknown. Overall, higher levels of moral distress were consistently associated with an increased likelihood of considering leaving the current position.

The participant flow is presented in Figure 1. Of the participants, 136 worked in specialist care and 75 in primary care. Participant characteristics are presented in Table 1. Approximately 15% of participants reported having previously left a position due to moral distress.

**Figure 1:**
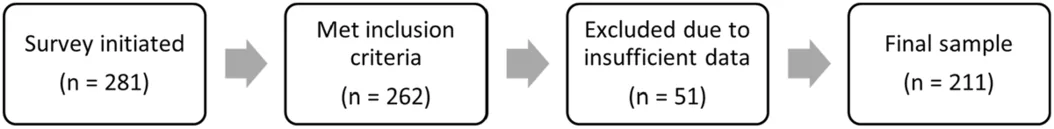
Flowchart of participant inclusion. The figure shows the number of participants assessed for eligibility, excluded and included in the study population.

**Table 1:** Participant characteristics by healthcare context in the study population (*n* = 211).

Participant characteristics	Primary care *n* (%)	Specialist care *n* (%)
Age group (total *n* = 211)		
20–29 years	6 (8.0)	22 (16.2)
30–39 years	27 (36.0)	49 (36.0)
40–49 years	25 (33.3)	26 (19.1)
50–59 years	9 (12.0)	29 (21.3)
≥ 60 years	8 (10.7)	10 (7.4)
Sex (total *n* = 209)		
Women	69 (94.5)	132 (97.1)
Men	4 (5.5)	3 (2.2)
Other/Prefer not to say	0 (0.0)	1 (0.7)
Years of professional experience (total *n* = 211)		
< 1	3 (4.0)	4 (2.9)
1–5	13 (17.3)	33 (24.3)
6–10	17 (22.7)	24 (17.6)
11–20	26 (34.7)	38 (27.9)
≥ 21	16 (21.3)	37 (27.2)
Education level (total *n* = 210)		
Bachelor's degree	57 (76.0)	92 (68.1)
Postgraduate diploma	13 (17.3)	24 (17.8)
Master's degree	1 (1.3)	10 (7.4)
Doctoral degree	4 (5.3)	9 (6.7)
Patient groups		
Children (0–17 years)	50 (66.7)	41 (30.1)
Adults (18–64 years)	69 (92.0)	108 (79.4)
Older adults (≥ 65 years)	71 (94.7)	98 (72.1)
Experience of leaving a position due to moral distress (total *n* = 185)		
No, never considered leaving or left	37 (56.9)	64 (52.5)
Yes, considered leaving but did not leave	19 (29.2)	39 (32.0)
Yes, left a position	9 (13.8)	19 (15.6)
Currently considering leaving a position (total *n* = 183)		
No	54 (85.7)	95 (79.2)
Yes	9 (14.3)	25 (20.8)

*Note:* Participants could report contact with more than one patient group. Percentages are based on available data for each variable; therefore, the number of observations (*n*) may vary due to missing data.

### 3.1 Levels of Moral Distress

Overall, the median total moral distress score was 71 (IQR 44–113). Median scores for the subscales were 29.0 (16.0–47.0) for system‐level, 11.0 (6.0–19.0) for patient‐level, 7.0 (2.0–16.0) for team/staff‐level and 18.0 (11.0–31.0) for team/patient‐level moral distress. Standardised subscale scores differed significantly across the four dimensions of moral distress (Friedman χ^2^ (3) = 240, *p* < 0.001), with system‐level moral distress showing the highest standardised scores and team/staff‐level moral distress the lowest.

The subscales and the five highest‐ranked items are presented in Figures 2 and 3. Free‐text responses (*n* = 32) reflected similar patterns. Participants described concerns related to resource constraints and organisational conditions, difficulties in prioritising patients and allocating responsibility for care, and situations where care was perceived to be compromised or not aligned with their professional judgement. Examples included long waiting times, limited access to dietetic care, unclear responsibility when patients were transferred between services and situations where decisions made by other professionals conflicted with dietetic assessments (see Table S2).

**Figure 2:**
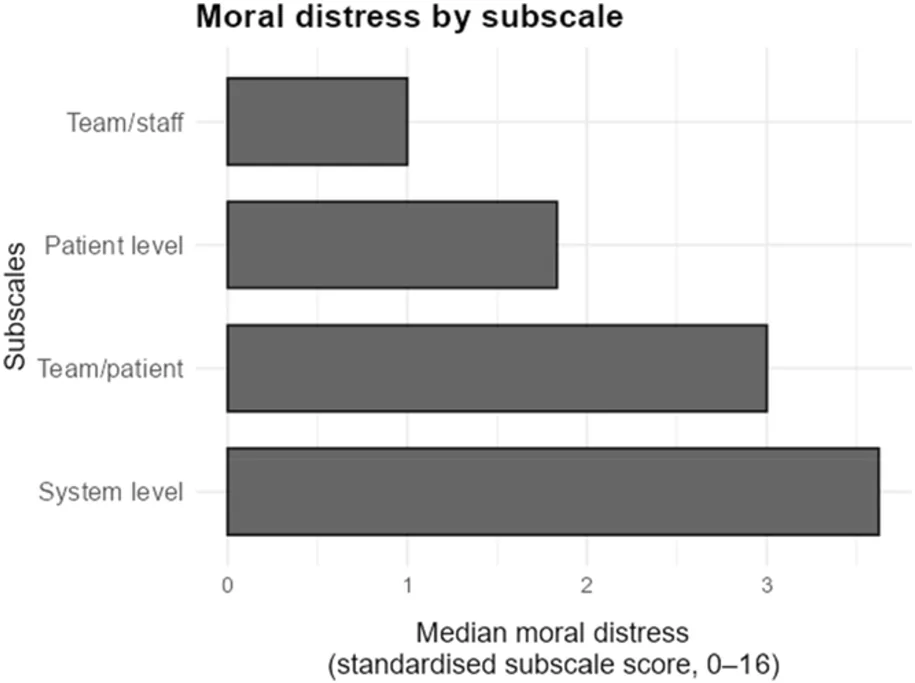
Moral distress by subscale. Median values (0–16) are shown for standardised subscale scores, calculated based on available data.

**Figure 3:**
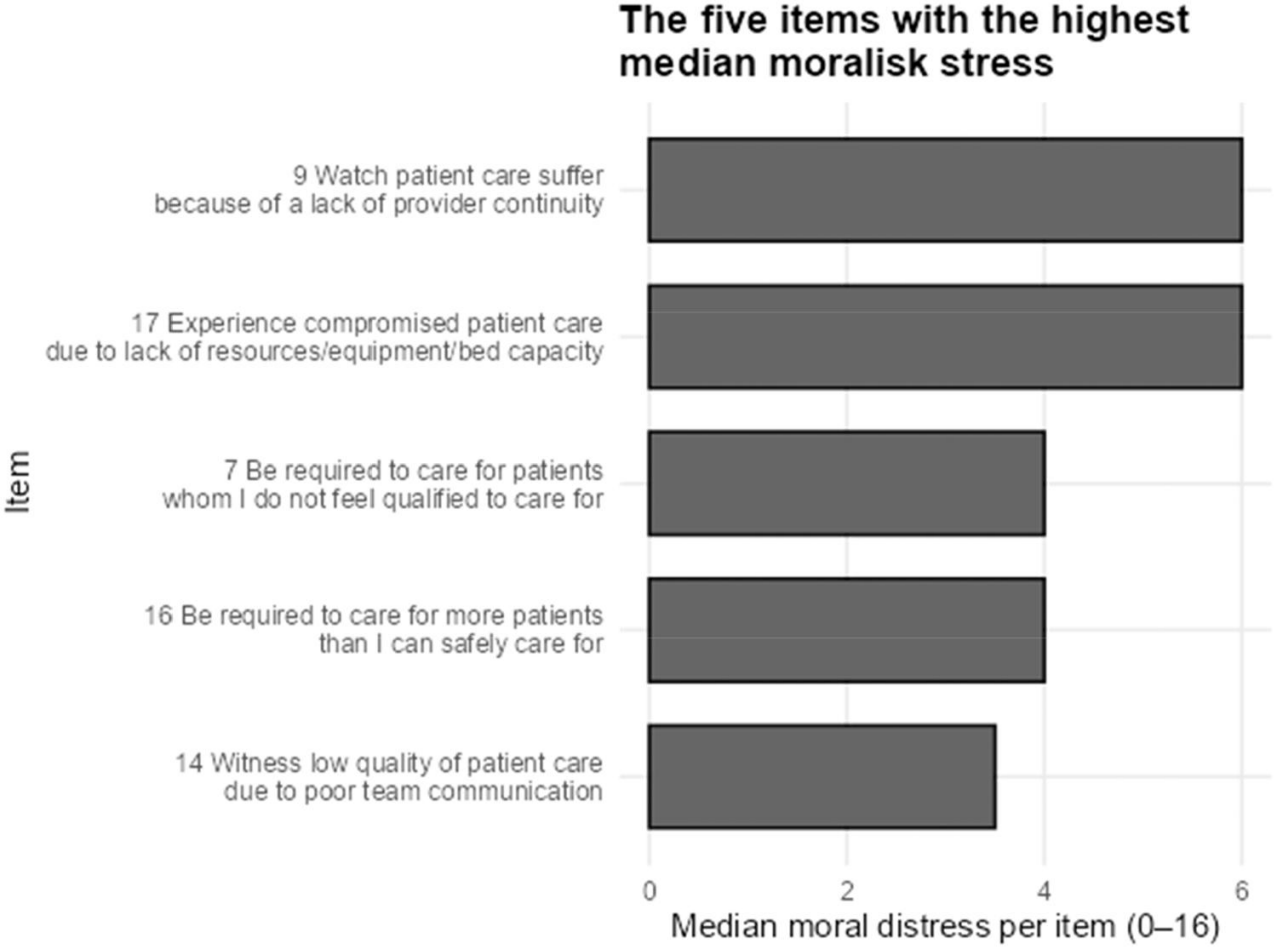
The five items with the highest levels of moral distress. The figure shows median values (0–16) for each item, calculated based on available data.

### 3.2 Differences in Moral Distress Between Primary Care and Specialist Care

No statistically significant difference in total moral distress was observed between primary and specialist care. Higher levels of team/patient‐related moral distress were observed among RDs in specialist care, although the effect size was small (*r* = −0.20). Missing data at the scale level ranged from 0.5% to 12.3% (Table 2). Item‐level missingness generally increased towards the end of the questionnaire, although some variation across individual items was observed.

**Table 2:** Moral distress by healthcare context (median [Q1–Q3]) (Mann–Whitney *U*).

Variable	*n*	Primary care	*n*	Specialist care	*U*	*p*	*r*	Missing *n* (%)
Total score	63	73.0 (39.0–110)	122	71.0 (47.3–113)	3546	0.390	−0.08	26 (12.3)
System	67	33.0 (14.5–52.5)	128	28.0 (16.8–40.3)	3965	0.388	0.08	16 (7.6)
Patient	75	10.0 (5.5–17.0)	135	12.0 (6.0–20.5)	4562	0.235	−0.10	1 (0.5)
Team/staff	64	7.0 (1.75–13.5)	125	7.0 (3.0–18.0)	3672	0.356	−0.08	22 (10.4)
Team/patient	65	16.0 (8.0–27.0)	127	20.0 (11.5–36.0)	3305	0.024	−0.20	19 (9.0)

*Note: p*‐values refer to moral distress. Missing data are reported relative to the study population (*n* = 211). *n* varies according to the availability of complete data for each score.

### 3.3 Association Between Moral Distress and Current Intention to Leave a Position

Participants who reported considering leaving their current position had higher median levels of moral distress than those who did not (Table 3).

**Table 3:** Total moral distress by current intention to leave a position (median [Q1–Q3]).

Intention to leave	*n* (%)	Median (Q1–Q3)
No	149 (81.4)	62 (39–98)
Yes	34 (18.6)	120 (89–152)

*Note:* Moral distress refers to the total score (range 0–432). *n* = 183.

The association between total moral distress and current intention to leave a position was analysed using binary logistic regression (Table 4).

**Table 4:** The association between moral distress and intention to leave a position (logistic regression).

Predictor	Model 1: Crude OR (95% CI)	*p*	Model 2: Adjusted OR (95% CI)	*p*
Total moral distress	1.016 (1.008–1.023)	< 0.001	1.016 (1.008–1.023)	< 0.001

*Note:* Model 1 = unadjusted model (*n* = 183). Model 2 = adjusted for healthcare context, years of professional experience and level of education (*n* = 182). The adjusted model included 4 predictors and 34 events (participants reporting intention to leave), corresponding to approximately 8.5 events per variable.

Abbreviations: CI, confidence interval; OR, odds ratio.

Higher levels of total moral distress were associated with an increased likelihood of considering leaving the current position (OR = 1.016, 95% CI 1.008–1.023, *p* < 0.001), both in the unadjusted model and after adjustment for healthcare context, education level and years of professional experience.

The overall model was statistically significant (likelihood‐ratio χ^2^ = 23.4, df = 4, *p* < 0.001) and showed acceptable discrimination (AUC = 0.780). The Box‐Tidwell test indicated some deviation from the assumption of linearity in the logit (*p* = 0.029).

### 3.4 Psychometric Properties of the MMD‑HP in a Sample of RDs

The factor structure of the MMD‑HP was examined using confirmatory factor analysis based on all available item‑level data from participants who met the inclusion criteria (*n* = 262). Model fit indices were RMSEA = 0.077 (90% CI 0.070–0.084), CFI = 0.79 and TLI = 0.77. Internal consistency was α = 0.92 for the total scale and ranged from α = 0.75 to α = 0.83 for the subscales.

## 4 Discussion

The present study contributes to a limited body of research examining moral distress among RDs. Levels of moral distress were broadly similar across healthcare contexts, while system‐level moral distress showed the highest standardised scores among the four dimensions of the MMD‐HP. Higher levels of moral distress were associated with an increased likelihood of considering leaving the current position.

The Swedish validation study of the MMD‐HP reported a mean moral distress score of 67.8 among a heterogeneous sample of healthcare professionals [31], whereas substantially higher levels have been reported in other healthcare populations, including intensive care nurses, with a mean score of 142.4 [40]. In the present study, the median moral distress score was 71. Direct comparisons should be interpreted with caution, as studies differ in terms of populations and reporting practices, with some reporting means and standard deviations and others reporting medians and interquartile ranges. Nevertheless, the reported levels do not appear markedly different from those previously reported in Swedish healthcare populations.

The highest‐ranked MMD‐HP items in the present study primarily related to lack of continuity of care, resource constraints, requirements to care for more patients than was perceived to be safe and poor team communication. While this pattern is broadly consistent with previous research identifying resource constraints, workload and communication‐related issues among the most prominent sources of moral distress across healthcare professions [42], other studies have reported end‐of‐life treatment decisions, such as the continuation of aggressive treatment despite limited patient benefit, as the most highly ranked sources of moral distress [40]. The pattern observed among RDs may therefore suggest that moral distress is more closely related to challenges in the organisation and coordination of care than to end‐of‐life treatment decisions, which have been identified as prominent sources of moral distress in some other healthcare populations.

Higher levels of total moral distress were associated with intention to leave the current position. This association remained after adjustment for healthcare context, education level and professional experience.

Previous studies have similarly reported higher levels of moral distress among healthcare professionals considering leaving their position compared with those who were not [40, 41, 43]. Despite differences in professional groups, care settings and study populations, the association between moral distress and intention to leave appears to be relatively consistent across studies. Previous research has also linked moral distress to burnout, emotional exhaustion and job dissatisfaction [44, 45, 46], suggesting that the implications of moral distress may extend beyond turnover intentions and may also affect healthcare professionals' wellbeing and sustainability in practice. Similar retention challenges have been reported across allied health professions, including dietetics, where workload, burnout, organisational conditions, professional support, management and career development opportunities have been identified as important influences on intentions to leave the profession [47]. This suggests that turnover intention among dietitians is likely influenced by multiple interacting individual and organisational factors rather than by moral distress alone.

Of the four MMD‐HP dimensions, system‐level moral distress showed the highest standardised scores. This was also reflected in the free‐text responses, where many of the situations described related to organisational aspects of care, including resource constraints, waiting times, prioritisation, unclear allocation of responsibility and patients at risk of falling between services or levels of care. This is consistent with previous research in which resource constraints, poor ethical climate and organisational barriers to providing good care have been identified as important contributors to moral distress [48, 49]. Moral distress was originally conceptualised as the distress that may arise when healthcare professionals are repeatedly constrained from acting in accordance with what they perceive to be ethically appropriate [20, 21]. The pattern observed in the present study is consistent with the view that moral distress is an individual experience that often arises in response to organisational and systemic constraints [49].

In dietetic practice, organisational constraints may include limited access to dietetic services, challenges related to continuity of care, resource allocation and collaboration across healthcare services [15]. Similar organisational factors have also been linked to dietitians' job satisfaction and staff retention [50]. These findings suggest that moral distress may be relevant to turnover intentions among RDs.

Similar to previous research using the MMD‐HP [51], no statistically significant differences in total moral distress were observed between RDs working in primary and specialist care. Although higher team/patient‐related moral distress was observed among RDs working in specialist care, the effect size was small. Furthermore, given the exploratory nature of the subscale analyses, the absence of adjustment for multiple testing, and the limited support for the four‐factor structure of the MMD‐HP in the present sample, findings related to individual subscales should be interpreted cautiously.

The absence of differences in total moral distress does not necessarily imply that the ethical challenges encountered in primary and specialist care are the same. As the MMD‐HP captures a specific set of situations previously identified as sources of moral distress [30], some context‐specific ethical challenges relevant to dietetic practice may not be fully reflected in the instrument. This may be particularly relevant given the limited representation of RDs in previous moral distress research.

To our knowledge, no previous quantitative study has examined moral distress and its association with intention to leave among RDs. The results provide new insights into the levels and dimensions of moral distress within the dietetic profession.

### 4.1 Methodological Discussion

A limitation of this study is its cross‐sectional design, which allows associations between variables to be examined but precludes conclusions about causality. For example, although moral distress was associated with intention to leave the current position, it is not possible to determine the direction of this relationship. Higher levels of moral distress may contribute to intentions to leave, but it is also possible that individuals who are considering leaving their position perceive or report higher levels of moral distress. Longitudinal studies are needed to examine these relationships over time.

The number of participants varied across analyses due to item non‐response. Missing values were not imputed; instead, the analytical sample was reported for each analysis. This approach increases transparency and avoids introducing assumptions about the missing data that could influence the results. The observed pattern of item non‐response suggests that survey attrition may have contributed to missing data, although item‐specific factors cannot be ruled out. Furthermore, no substantial differences were observed between participants included and excluded from the primary analyses with respect to healthcare context, age, sex, education level or years of professional experience.

The adjusted logistic regression model included four predictors and was estimated using a sample of 182 participants. In the logistic regression model, 34 participants reported considering leaving their current position, corresponding to approximately 8.5 events per variable. The association between moral distress and intention to leave remained stable across alternative model specifications with fewer predictors, providing some support for the robustness of the observed association despite the relatively limited number of events.

Additional logistic regression diagnostics suggested that the assumption of linearity in the logit may not have been fully satisfied. However, the proportion of participants reporting intention to leave was higher at successively higher quartiles of moral distress, increasing from 4.3% in the lowest quartile to 39.1% in the highest quartile. This suggests a positive association between total moral distress and intention to leave across the full range of scores, although the rate of increase may not be perfectly linear. The logistic regression findings should therefore be interpreted with some caution.

### 4.2 Sampling and Generalisability

The study was based on a convenience sample. As the survey was distributed through multiple channels and the number of eligible individuals who received the survey was unknown, a response rate could not be calculated. The possibility of self‐selection bias cannot be excluded. For example, RDs with a particular interest in ethics or experience of moral distress may have been more likely to participate, which could have resulted in an overestimation of moral distress. Conversely, individuals experiencing high workloads, limited time or substantial moral distress may have been less likely to complete the survey, which could have contributed to an underestimation. The direction and magnitude of any potential self‐selection bias therefore remain uncertain.

A methodological consideration is the use of the MMD‐HP in a professional group that has been largely absent from both the development and validation of the instrument. Although the MMD‐HP has been validated in several countries [31, 34, 37, 52], the instrument has primarily been developed and tested in samples of healthcare professionals in which RDs have rarely been represented as a distinct professional group [30]. Consequently, limited studies are available regarding the use of the instrument among RDs. Therefore, cognitive interviews were conducted prior to data collection. Participants suggested that some items varied in perceived relevance across areas of practice and healthcare contexts, but no concerns were identified that indicated the instrument was unsuitable for use among RDs. The findings informed revisions to introductory texts, instructions and background questions, while the original MMD‐HP items were retained unchanged.

The psychometric tests provided additional information about the use of the MMD‐HP among RDs. Internal consistency was high for the total scale (α = 0.92) and acceptable to good for the subscales (α = 0.75–0.83). However, CFA provided weaker support for the established four‐factor model than previously reported in the Swedish validation study [31]. Although the present study was not designed as a psychometric validation study, the findings may nevertheless be informative. Given the limited representation of RDs in previous moral distress research and in studies evaluating the MMD‐HP, the weaker model fit may suggest that the established factor structure does not fully capture how MMD‐HP items relate to one another among RDs.

This interpretation is consistent with the findings from the cognitive interviews, where participants described variation in the relevance of some items across practice areas and healthcare contexts, and with the prominence of organisational and system‐related concerns in the present study. Further research is needed to examine the factor structure and validity of the MMD‐HP among RDs. Until then, findings related to individual subscales should be interpreted cautiously.

## 5 Conclusion

Higher levels of moral distress were associated with intention to leave the current position. System‐level moral distress showed the highest standardised scores among the four dimensions of the MMD‐HP, suggesting that organisational and system‐related factors may be important contributors to dietitians' experiences of moral distress. No statistically significant differences in total moral distress were observed between primary and specialist care. The findings contribute to the limited literature on moral distress among RDs and suggest that moral distress may be relevant not only as an ethical issue but also in relation to turnover intentions among RDs.

## Author Contributions

**Susanna Pohjola:** conceptualisation, methodology, investigation, data curation, formal analysis, writing – original draft preparation, writing – review and editing. **Åsa Grauman:** conceptualisation, methodology, supervision, writing – review and editing. **Anna T. Höglund:** conceptualisation, methodology, supervision, writing – review and editing. **Elin Lövestam:** conceptualisation, methodology, funding acquisition, supervision, writing – review and editing. All authors contributed to interpretation of the findings and approved the final manuscript.

## Ethics Statement

The study was approved by the Swedish Ethical Review Authority (reference number 2023‐03151‐01 and amendment 2026‐00124‐02).

## Conflicts of Interest

The authors declare no conflicts of interest.

## Supporting information


Supporting File 1

Supporting File 2

## Data Availability

The data supporting the findings of this study are available from the corresponding author upon reasonable request.
