# Supplementary material for: Moral Distress and Intention to Leave Current Position Among Registered Dietitians: A Cross‐Sectional Study
Source: J Hum Nutr Diet. 2026 Oct 25;39(5):e70368. doi: 10.1111/jhn.70368 (PMC13696964; doi:10.1111/jhn.70368)
Supplement: Supplementary file 1 [file JHN-39-0-s001.docx]

| Supplementary Table S2. Free-text responses describing additional situations giving rise to moral distress | | | |
| --- | --- | --- | --- |
| Participant | Situation 1 (frequency×intensity) | Situation 2 (frequency×intensity) | Situation 3 (frequency×intensity) |
| 1 | prioritising patients *(prioritering av patienter)* (4x2) | - | - |
| 2 | safeguarding reports *(orosanmälningar)* (2×3) | unclear treatment responsibilities *(otydlighet i vilken behandlingsinsats vi ska ge)* (2×2) | limited insight and managerial support *(för lite insyn och stöd från chef)* (2×3) |
| 3 | patients receiving harmful dietary advice from physicians *(patienter som fått kostråd av läkare, som är direkt skadliga för patienten)* (3×4) | situations where assistant nurses or nurses provide too little food and snacks to a patient with severe obesity, considering that the patient needs to lose weight, which is completely against all evidence that an inpatient with illness should not receive sufficient energy *(situationer när undersköterskor eller sjuksköterskor ger för lite mat och mellanmål till patient med kraftig obesitas, med tanke att patienten behöver gå ner i vikt, vilket är helt emot all evidens att en inneliggande patient med sjukdom inte skulle behöva tillräckligt med energi)* (2×3) | - |
| 4 | there is a lack of dietitian resources within, for example, CAMHS in Region X. There is a lack of knowledge and active work with selective eating in habilitation services and in the region there is also no one responsible for selective eating among other patients and no structured management of ARFID *(De saknas dietistresurser inom tex BUP i region X. Saknas kunskap/aktivt arbete med selektivt ätande på HAB och i regionen är de också ingen som har ansvaret för selektivtet i hos övriga patienter och ingen strukturerat omhändertagande om ARFID)* (4×4) | - | - |
| 5 | having to end contact with a patient despite the patient wanting continued contact *(När man måste avsluta kontakt med en patient trots att hen vill ha fortsatt kontakt.)* (2×3) | - | - |
| 6 | long waiting times for follow‑up appointments where the dietitian needs to prioritise which patient should receive care first *(långa väntetider för återbesök där det ligger på dietist att prioritera vem som ska få vård först)* (4×4) | requirement to transfer responsibility for treatment to a healthcare provider without the appropriate knowledge *(krav på att ge över ansvar av behandling på vårdgivare utan rätt kunskap)* (3×4) | - |
| 7 | when preventive meal work does not work and the patient becomes undernourished. This creates stress in two ways, both because the patient experiences suffering and because I then need to initiate medical nutritional treatment to compensate for the inadequate meal work *(När det förebyggande måltidsarbetet inte fungerar så att patienten blir undernärd. Det stressar på två sett, dels för att patienten får ett lidande och dels för att jag då behöver sätta in medicinsk nutritionsbehandling för att kompensera för det bristande måltidsarbetet.)* (4×4) | - | - |
| 8 | when my employer’s organisation means that certain patient groups in need of nutritional care have no possibility to access it *(När min arbetsgivares organisation gör att vissa patientgrupper i behov av nutritionsvård inte har någon möjlighet att få det)* (4×4) | - | - |
| 9 | when other professions promise prescription of nutritional supplements but I as a dietitian assess that they are not needed *(När andra yrkesgrupper utlovar förskrivning av näringsdryck men jag som dietist bedömer att de inte behövs.)* (3×4) | - | - |
| 10 | patients who are too healthy for specialist care but too ill for primary care, where primary care takes responsibility simply because no one else wants to take on the case/patient *(Patienter som är för friska för specialiståvården men för sjuka för primärvården där primärvården tar på sig ansvaret bara för att ingen annan vill ta ärendet/patienten.)* (4×4) | - | - |
| 11 | when patients are sent back and forth between primary and specialist care and no one takes responsibility for the case. The dietitian who tries to support nutritional problems stands alone, sometimes being the only consistent contact while others “close the door” *(När patienter skickas runt primärvård/specialistvård och ingen vill ha hand om ärendet. Dietist som försöker stötta med nutritionsproblematik står ensam, ibland den enda fasta kontakten medan alla andra "stänger dörren")* (4×4) | - | - |
| 12 | a patient wants prescription of oral nutritional supplements but shows no results from it. Because the patient is thin/vulnerable it becomes difficult to assess whether one can/should discontinue after trying it out since the patient feels safer with it or experiences improvement *(Patient vill ha förskrivning kosttillägg, men visar inte resultat av det. P g a mager/sårbar svårt att bedöma om man kan/bör ta bort efter test p g a patient känner sig tryggare med eller upplevelse av att må bättre)* (2×2) | referrers writing in referrals “prescription of nutritional supplements” and sometimes having already suggested it to the patient, which may reduce motivation to work with fortified diet *(Remittenter skriver i remiss "förskrivning näringsdryck" och ibland redan föreslagit för patient som kan bli mer omotiverad att arbeta med berikad kost i första hand.)* (4×3) | many different systems that are often limited/poorly designed, time‑consuming and mentally fragmenting *(Många olika system som ofta är begränsade/dåligt uppbyggda, tidsödande och blir splittrad mentalt.)* (4×4) |
| 13 | when the patient’s relatives/parents have their own views on how best to care for their relatives, which go against my clinical judgement *(När patientens närstående/ föräldrar har egna åsikter om hur de bäst tar hand om sina närstående som dock går emot min kliniska bedömning.)* (1×4) | - | - |
| 14 | lack of dietitian resources – what I do not have time to do, no one else will do regardless of how urgent the case is *(Resursbrist på specifikt dietistresurs - det jag inte hinner göra, kommer ingen annan göra oavsett hur angeläget ärendet är.)* (4×4) | treatment has an uncertain outcome, meaning in cases where NO ONE CAN KNOW whether treatment of a severely ill patient will have a good outcome, but where the treatment also entails suffering for the *patient (Behandling har en osäker utgång, dvs i de fall INGEN KAN veta om behandling av svårt sjuk kan ha gott resultat men där behandlingen också innebär lidande för patienten.)* (3×3) | - |
| 15 | the patient needs to come in person even if a telephone consultation would be better, because reimbursement is higher for in‑person visits *(Patienten behöver komma på plats även om det är bättre telefonsamtal på grund av ersättning är bättre på plats.)* (1×2) | minors are denied treatment due to an invalid LMA card *(Underåriga får inte behandling på grund av ogiltig LMA-kort)* (1×3) | - |
| 16 | providing a patient with a larger prescription of nutritional supplements than covered by the co‑payment due to difficult financial circumstances *(Ge patient större förskrivning av kosttillägg än egenavgiften avser (pga av svåra ekonomiska omständigheter hos patienten))* (4×3) | instructions from management to register visits with incorrect time codes for greater (but incorrect) reimbursement from the region *(Instruktioner från ledning av registrera besök med felaktiga tidskoder för större (men felaktig) ersättning från regionen)* (3×2) | large amounts of administrative care work required for patients but not supported by the employer or reimbursed by the region (e.g. contacting social services/family/home care/other providers; creating feeding plans; supporting relatives in nutritional treatment at home) *(Stora mängder adminstrativt vårdarbete som patienter kräver men som ej ges utrymmes av arbetsgivaren eller ersättning från regionen (ex ringa och samordna biståndshandläggare/anhöriga/hemtjänst/andra vårdgivare; göra sondmatsscheman/matscheman; stötta anhöriga i nutritionsbehandlingen i hemmet))* (4×4) |
| 17 | not feeling sufficiently knowledgeable to treat a patient group but having to do so anyway due to lack of time for reading *(Att inte känna sig kunnig nog för att behandla en patientgrupp, men måste göra det ändå pga bristande tid för inläsning)* (1×4) | - | - |
| 18 | having to prioritise among patients who all need help from a dietitian *(Att prioritera bland patienter som alla behöver hjälp av dietist.)* (2×2) | - | - |
| 19 | unclear when the patient will be discharged from the ward, but in need of nutritional prescription at discharge; however, prescriptions should not be made if discharge does not occur or needs change, and urgent orders should not be made according to guidelines *(oklart när patienten ska hem från avdelningen, men är i behov av förskrivning VID hemgång/ att den finns när patienten kommit hem. Bör dock heller inte göra förskrivning om patienten inte ska hem alt behov förändras. Bör dessutom ej göra akutbeställningar enligt x.)* (4×4) | - | - |
| 20 | experiencing stress when a colleague (dietitian) was on sick leave and I could not manage all patients from their waiting list who needed to see a dietitian urgently or for planned follow‑up *(Jag upplevde stress när min kollega (dietist) var sjukskriven och jag kunde inte ta alla patienter från hennes väntelista som behövde träffa dietist snarast elle råd sin planerade uppföljning)* (3×4) | - | - |
| 21 | having to say no to treating patients because the diagnosis/problem is not within one’s assignment, while no other profession has responsibility for that patient group, leaving the patient without care *(När man måste säga nej till att vårda patienter pga diagnosen/problemet ej ingår i ens uppdrag, men det finns ingen annan som har patientgruppen i deras uppdrag heller, så patienten står utan vård helt och hållet.)* (4×4) | - | - |
| 22 | a dietitian colleague lacking competence and no one addressing it *(När dietistkollega har bristande kompetens och ingen bryr sig)* (1×2) | a dietitian colleague lacking engagement affecting patients *(När dietistkollega har bristande intresse vilket går utöver patienter)* (3×3) | a dietitian colleague lacking competence and not wanting to improve *(När dietistkollega har bristande kompetens men inte vill lära sig)* (3×3) |
| 23 | pressure to prescribe oral nutritional supplements because other healthcare professionals have recommended it *(Press att förskriva kosttillägg för att annan vårdpersonal rekommenderat detta)* (1×2) | pressure to prescribe nutritional supplements for social/economic reasons rather than medical ones *(Press att förskriva kosttillägg av sociala/ekonomiska skäl snarare än medicinska)* (1×3) | - |
| 24 | assessments related to safeguarding reports for children *(Bedömningar kring orosanmälningar för barn)* (3×4) | difficulties collaborating with other services, for example school, preschool, child health services, habilitation *(Svårigheter att samarbeta med andra instanser, t ex skola, förskola, BVC, habilitering)* (4×4) | - |
| 25 | in primary care, having to maximise the number of patients despite feeling that there is no time *(Inom Primärvården att maximera antal patienter fastän känna att tid inte fanns.)* (4×4) | prescribing nutritional drinks to patients who may not actually need them *(Förskriva näringsdrycker till patienter som kanske egentligen inte behöver (primärvård).)* (4×4) | registering visits as longer to receive more reimbursement even though the actual time was shorter *(Registrera besök som längre tid för att få mer ersättning fastän det ej tog den tiden i verkligheten (primärvård).)* (3×4) |
| 26 | prescription of nutritional drinks for economic reasons *(Förskrivning på ND av ekonomiska skäl)* (4×3) | - | - |
| 27 | having to make decisions regarding patients’ financial situations, for example charging for missed visits or deciding on costs for oral nutritional supplements *(När jag behövt ta beslut som rör patienternas ekonomiska situation (t ex skicka räkning för uteblivet besök) eller besluta om kostnad för kosttillägg)* (3×3) | - | - |
| 28 | too few resources and too many referrals *(För lite resurser och många remisser in)* (1×2) | - | - |
| 29 | routinely transitioning to palliative care without offering patients appropriate treatment *(När man mer rutinmässigt övergår till palliativ vård och inte erbjuder patienter relevant behandling.)* (3×4) | - | - |
| 30 | prioritising patients on my waiting list against each other, knowing that some may wait over a year for follow‑up when they should receive it within three months *(prioritera patienter i min väntelista mot varandra, med vetskap om att vissa får vänta över ett år på återbesök som borde få det inom 3 månader)* (4×4) | trying to prioritise my patient groups against colleagues’ patient groups when everyone has a high workload and is under stress *(försöka prioritera mina patientgrupper mot kollegors patientgrupper, när alla har mycket att göra och är stressade)* (3×4) | - |
| 31 | having to refuse care requests from patients or relatives because the diagnosis or nutritional problem falls outside the dietitian’s (limited) remit, despite knowing the patient could benefit from dietetic care *(När jag behövt avvisa vårdbegäran från patienter eller närstående då diagnos eller nutritionsproblem inte faller inom ramen för dietistens (begränsade) uppdrag, trots att jag vet att patienten skulle kunna haft nytta av dietistkontakt.)* (2×2) | having to refer nutritional measures or assessments to other healthcare professionals due to agreements on responsibility, meaning patients risk receiving poorer care than if managed by a dietitian *(Att behöva hänvisa vissa nutritionsåtgärder eller bedömningar till annan vårdpersonal på grund av överenskommelser om ansvar för nutritionsbehandling som innebär att andra professioner ska göra sådant som egentligen kräver dietistkompetens, dvs patienten riskerar att få sämre vård än om en dietist skulle sköta behandlingen.)* (3×3) | - |
| 32 | having to adapt what is prescribed in terms of type and quantity of oral nutritional supplements or tube feeding due to regulations, meaning the patient’s best interest cannot be prioritised *(Att behöva anpassa vad man förskriver för typ och antal av kosttillägg/sondmat pga regler kring detta som vi ej kan påverka, gör att patientens bästa ej går att ha som nr 1)* (3×3) | when a patient is discharged from a ward without being able to maintain adequate intake of fluids and energy *(När en patient skickas hem från vårdavdelning utan att kunna försörja sig vad gäller vätska och energi)* (2×4) | - |
| Note. English translations are provided alongside the original Swedish wording. | | | |
