# Supplementary material for: Moral Distress and Intention to Leave Current Position Among Registered Dietitians: A Cross‐Sectional Study
Source: J Hum Nutr Diet. 2026 Oct 25;39(5):e70368. doi: 10.1111/jhn.70368 (PMC13696964; doi:10.1111/jhn.70368)
Supplement: Supplementary file 2 [file JHN-39-0-s002.docx]

STROBE Statement—Checklist of items that should be included in reports of ***cross-sectional studies***

|  | Item No | Recommendation | Comments |  |
| --- | --- | --- | --- | --- |
| **Title and abstract** | 1 | (*a*) Indicate the study’s design with a commonly used term in the title or the abstract | Reported in title as a cross-sectional study. |  |
|  |  | (*b*) Provide in the abstract an informative and balanced summary of what was done and what was found | Addressed in abstract (Background, Methods, Results, Conclusion). |  |
| Introduction | | |  |  |
| Background/rationale | 2 | Explain the scientific background and rationale for the investigation being reported | Addressed in Introduction. |  |
| Objectives | 3 | State specific objectives, including any prespecified hypotheses | Study aims and research questions stated in Introduction. |  |
| Methods | | |  |  |
| Study design | 4 | Present key elements of study design early in the paper | Described under Study design. |  |
| Setting | 5 | Describe the setting, locations, and relevant dates, including periods of recruitment, exposure, follow-up, and data collection | Setting, recruitment period, and data collection dates described under Participants, recruitment, and data collection. |  |
| Participants | 6 | (*a*) Give the eligibility criteria, and the sources and methods of selection of participants | Eligibility criteria, recruitment, and participant selection described under Participants, recruitment, and data collection. |  |
| Variables | 7 | Clearly define all outcomes, exposures, predictors, potential confounders, and effect modifiers. Give diagnostic criteria, if applicable | Outcomes, predictors, and covariates described under Measures and Data analysis. |  |
| Data sources/ measurement | 8* | For each variable of interest, give sources of data and details of methods of assessment (measurement). Describe comparability of assessment methods if there is more than one group | Data sources and measurement methods described under Measures. |  |
| Bias | 9 | Describe any efforts to address potential sources of bias | Potential sources of bias discussed under Sampling and generalisability and Methodological discussion. |  |
| Study size | 10 | Explain how the study size was arrived at | No formal sample size calculation was performed. Recruitment was extended when dietitians working in primary care were underrepresented, to ensure a sufficient basis for planned comparisons between primary and specialist care. |  |
| Quantitative variables | 11 | Explain how quantitative variables were handled in the analyses. If applicable, describe which groupings were chosen and why | Quantitative variables and score calculations described under Measures and Data analysis. |  |
| Statistical methods | 12 | (*a*) Describe all statistical methods, including those used to control for confounding | Statistical methods, including adjustment for potential confounders (care context, professional experience and education level), described under Data analysis. |  |
|  |  | (*b*) Describe any methods used to examine subgroups and interactions | Subgroup analyses by healthcare context and sociodemographic variables described under Data analysis. |  |
|  |  | (*c*) Explain how missing data were addressed | Missing data handled through listwise deletion; approach described under Data analysis. |  |
|  |  | (*d*) If applicable, describe analytical methods taking account of sampling strategy | Not applicable; no complex sampling strategy was employed. |  |
|  |  | (*e*) Describe any sensitivity analyses | No formal sensitivity analyses performed. |  |
| Results | | |  |  |
| Participants | 13* | (a) Report numbers of individuals at each stage of study—eg numbers potentially eligible, examined for eligibility, confirmed eligible, included in the study, completing follow-up, and analysed | Numbers included at each stage reported in Figure 1 and Results. |  |
|  |  | (b) Give reasons for non-participation at each stage | Partly addressed. Exclusions are described in the Methods section and presented in Figure 1. Reasons for non-response could not be assessed. |  |
|  |  | (c) Consider use of a flow diagram | provided in Figure 1. |  |
| Descriptive data | 14* | (a) Give characteristics of study participants (eg demographic, clinical, social) and information on exposures and potential confounders | Participant characteristics reported in Table 1. |  |
|  |  | (b) Indicate number of participants with missing data for each variable of interest | Missing data reflected through variable-specific sample sizes and reported n-values. |  |
| Outcome data | 15* | Report numbers of outcome events or summary measures | Outcome measures reported in Tables 2–5 and Figures 2–3. |  |
| Main results | 16 | (*a*) Give unadjusted estimates and, if applicable, confounder-adjusted estimates and their precision (eg, 95% confidence interval). Make clear which confounders were adjusted for and why they were included | Crude and adjusted estimates with 95% confidence intervals reported in Tables 3 and 5. |  |
|  |  | (*b*) Report category boundaries when continuous variables were categorized | Categorised variables and category boundaries reported in Table 1. |  |
|  |  | (*c*) If relevant, consider translating estimates of relative risk into absolute risk for a meaningful time period | Not applicable for this cross-sectional study. |  |
| Other analyses | 17 | Report other analyses done—eg analyses of subgroups and interactions, and sensitivity analyses | Additional analyses reported in Supplementary Tables S3 and S4 and psychometric analyses section. |  |
| Discussion | | |  |  |
| Key results | 18 | Summarise key results with reference to study objectives | Key findings summarised in the first paragraph of the Discussion. |  |
| Limitations | 19 | Discuss limitations of the study, taking into account sources of potential bias or imprecision. Discuss both direction and magnitude of any potential bias | Limitations discussed under Methodological discussion and Sampling and generalisability. |  |
| Interpretation | 20 | Give a cautious overall interpretation of results considering objectives, limitations, multiplicity of analyses, results from similar studies, and other relevant evidence | Interpretation provided in the Discussion and Conclusion. |  |
| Generalisability | 21 | Discuss the generalisability (external validity) of the study results | Generalisability discussed under Sampling and generalisability. |  |
| Other information | | |  |  |
| Funding | 22 | Give the source of funding and the role of the funders for the present study and, if applicable, for the original study on which the present article is based | Funding reported in the Funding/Acknowledgements section, including funder name, grant number, and statement on the funder's role. |  |

*Give information separately for exposed and unexposed groups.

**Note:** An Explanation and Elaboration article discusses each checklist item and gives methodological background and published examples of transparent reporting. The STROBE checklist is best used in conjunction with this article (freely available on the Web sites of PLoS Medicine at http://www.plosmedicine.org/, Annals of Internal Medicine at http://www.annals.org/, and Epidemiology at http://www.epidem.com/). Information on the STROBE Initiative is available at [www.strobe-statement.org](http://www.strobe-statement.org).
